# Nano-Liposomal Beetroot Phyto-Pigment in Photodynamic Therapy as a Prospective Green Approach for Cancer Management: In Vitro Evaluation and Molecular Dynamic Simulation

**DOI:** 10.3390/pharmaceutics16081038

**Published:** 2024-08-03

**Authors:** Doaa Abdel Fadeel, Maha Fadel, Abdullah Ibrahim El-Kholy, Ahmed A. El-Rashedy, Engy Mohsen, Marwa I. Ezzat, Marwa Y. Issa

**Affiliations:** 1 Pharmaceutical Nanotechnology Unit, Department of Medical Applications of Laser, National Institute of Laser Enhanced Sciences (NILES), Cairo University, Giza 12613, Egypt; mahafmali@niles.cu.edu.eg (M.F.);; 2 Chemistry of Natural and Microbial Products Department, National Research Center (NRC), Giza 12622, Egypt; ahmedelrashedy45@gmail.com; 3 Department of Pharmacognosy, Faculty of Pharmacy, Cairo University, Cairo 11562, Egypt; engy.mohsen@pharma.cu.edu.eg (E.M.); marwa.ezzat@pharma.cu.edu.eg (M.I.E.); marwa.issa@pharma.cu.edu.eg (M.Y.I.)

**Keywords:** photodynamic therapy, beetroot extract, green photosensitizers, liposomes, molecular dynamic simulation

## Abstract

Using plant extracts as photosensitizers in photodynamic therapy (PDT) represents a significant green approach toward sustainability. This study investigates beetroot juice (BRJ), betanin, and their liposomal formulations (Lip-BRJ, Lip-Bet) as photosensitizers in cancer PDT. BRJ was prepared, and its betanin content was quantified via HPLC. The p-nitrosodimethylaniline (RNO)/imidazole technique monitored the singlet oxygen formation. BRJ and betanin decreased the RNO absorbance at 440 nm by 12% and 9% after 45 min of irradiation, respectively. Furthermore, betanin interaction with Bcl-2 proteins was examined using binding free energy analysis and molecular dynamic simulation. The results revealed favorable interactions with ΔG values of −40.94 kcal/mol. Then, BRJ, betanin, Lip-BRJ, and Lip-Bet were tested as photosensitizers on normal (HEK 293) and human lung cancer (A549) cell lines. Irradiation significantly enhanced the cytotoxicity of Lip-Bet on HEK 293 cells (20% cell viability at 2000 µg/mL) and A549 cells (13% cell viability at 1000 µg/mL). For Lip-BRJ, irradiation significantly enhanced the cytotoxicity on HEK 293 cells at lower concentrations and on A549 cells at all tested concentrations. These results proved the positive effect of light and liposomal encapsulation on the anticancer activity of betanin and BRJ, suggesting the efficiency of liposomal beetroot pigments as green photosensitizers.

## 1. Introduction

Photodynamic therapy (PDT) is an effective, non-invasive strategy for treating various malignant and nonmalignant conditions. PDT is a photochemical interaction between a photoactive molecule (photosensitizer), light energy, and molecular oxygen in the tissue. When a photosensitizer absorbs the light energy, it gets excited to higher energy levels. Subsequently, it transfers this acquired energy to suitable substrates. This reaction finally results in the generation of reactive oxygen species (ROS) and free radicals (type I) or singlet oxygen (type II) that irreversibly devastate the cells [1,2,3]. The activity of the produced radicals or other ROS is confined to the site where they are produced because they have a very short half-life and cannot travel distantly inside the cells [4]. Thus, by suitable delivery of the photosensitizer to the target tissue and employing a light source of appropriate wavelength, the PDT can specifically destroy the diseased tissues, saving the healthy ones. The selectivity of PDT gives it a substantial advantage over other cancer treatment approaches.

The physicochemical properties of the photosensitizers are the primary factor that governs the efficacy of PDT. Various chemically synthesized photosensitizers are applied in the PDT; however, their applications still meet some challenges. In addition to their high cost, the chemically synthesized photosensitizers may be hazardous to the human body and may elicit complicated side effects. Therefore, in recent years, endeavors have been made to search for green photosensitizers derived from plants as they are characterized by their high bioavailability, high biodegradability, high abundance, low cost, low toxicity, and low immunogenicity [5]. However, isolating individual phytoconstituents from the whole extract is a complicated, costly process. Moreover, using plant extracts that contain complex phytoconstituents in cutting-edge therapy, like PDT, was reported to be beneficial over single chemical compounds due to the synergetic effect of the different phyto-constituents [6,7]. Therefore, exploring well-known and locally cultivated crude plant extracts as potential photosensitizers is thought to be an eco-friendly approach to accomplishing the goals of sustainable development, especially in developing countries.

Beetroot extract (*Beta vulgaris* L.) has gained popularity as a potential “functional food”. It is considered a nutritious vegetable that is rich in natural pigments and can be added to salads or used for making juices. It is rich in micro-nutrients, carbohydrates, fats, and bioactive phytoconstituents, including carotenoids, polyphenols, flavonoids, saponins, and betalains (the water-soluble pigments) [8,9]. These bioactive phytoconstituents are essential in treating several diseases, including cardiovascular diseases, cancer, diabetes, cerebrovascular diseases, and chronic respiratory diseases [10].

The main pigments in the beetroot extract are called betalains, which comprise betacyanins (red pigment) and betaxanthins (yellow pigment) [11]. Betacyanins are the most prevalent pigments that account for 75–90% of the total pigments in red beetroot. Betanin (betanidin 5-*O*-β-glucoside) and its epimer isobetanin are the main betacyanins in beetroot. The concentration of betacyanins is highest in the peel and gradually diminishes toward the root’s interior [12]. Betanin is mainly employed as a colorant agent in the food and beverage industries. Moreover, betanin was reported to possess several biomedical activities as an antioxidant, anti-inflammatory, and anticancer agent [13,14]. 

Nevertheless, the use of betanin in the biomedical field is limited due to its rapid degradation and high susceptibility to changes in the surrounding conditions such as temperature, pH, oxygen, enzymes, and light [15,16]. These drawbacks can be resolved by encapsulating betanin in an appropriate nanocarrier. 

Liposomes are biodegradable, well-tolerated vesicular lipid-based nanoparticles comprising a synthetic or natural phospholipid bilayer membrane and an aqueous core. In aqueous media, they are self-assembled into vesicular structures composed of one or more concentric layers with hydrophilic heads pointing toward the outside surfaces and hydrophobic tails. This unique structure allows them to be a potential nanocarrier system that can accommodate hydrophobic and hydrophilic drugs [1,17,18]. 

Encapsulating pharmaceuticals in liposomes has been proven to ameliorate their stability, bioavailability, biocompatibility, and absorption through biological barriers [15]. In addition, liposomes protect the drugs from external factors and prevent primordial destruction, providing maximum effect with minimal systemic toxicity [18].

Liposomes were reported to enhance the photodynamic effect of several photosensitizers such as curcumin, indocyanine green, hypericin, chlorin e6, temoporfin, and methylene blue against various types of cancers [1,4].

The isolated component, betanin, was previously incorporated in liposomes to improve its stability in gastric fluids. However, few studies reported the liposomal encapsulation of the crude beetroot extract. Dias et al. incorporated beetroot extract in liposomes to be used as a colorant for cooked ham meat [19].

In this contribution, we developed a HPLC-UV technique that provides a fast, simple, and accurate quantitative assessment of betalains in beetroot juice using betanin as a marker compound. Then, we investigated the efficacy of beetroot extract juice (BRJ), its active constituent betanin, and its liposomal formulations as potential photosensitizers on normal and cancer cell lines after being activated by a visible light source at 535 nm. Another innovative aspect of our study is the molecular dynamic simulation study and the binding free energy measurements that were performed to examine the mechanism of selective interaction of betanin with Bcl-2 proteins. The Bcl-2 family consists of antiapoptotic proteins that are overexpressed, allowing cancer cells to survive and develop resistance to traditional treatment [20]. One promising approach to cancer treatment is the inhibition of these prosurvival proteins. Therefore, they were investigated as possible pharmacological targets of betanin in our study.

To our knowledge, this is the first study that incorporates beetroot extract and betanin in liposomes to be tested for their photodynamic activity and binding affinity to Bcl-2 proteins. 

## 2. Materials and Methods

### 2.1. Materials

Standard betanin (red beetroot extract diluted with dextrin), L-α-phosphatidylcholine from soybean, p-nitrosodimethylaniline (RNO), and imidazole were purchased from Sigma Aldrich (St. Louis, MO, USA). Cholesterol is a product of Tekkim (Tekkim, Istanbul, Turkey). All HPLC and analytical-grade reagents were purchased from Sigma Aldrich (USA).

Reagents for cell culture were as follows: the DMEM cell culture media (Dulbecco’s modified Eagle’s), trypsin-EDTA, streptomycin-penicillin, and fetal bovine serum (FBS) were purchased from Lonza (Lonza, Verviers, Belgium). The MTT reagent [3-(4,5-dimethylthia-zol-2-yl)-2,5-diphenyl tetrazolium bromide] was purchased from BIO BASIC (BIO BASIC, Markham, ON, Canada). 

### 2.2. Plant Material and Preparation of the Beetroot Juice 

The roots of *B. vulgaris* were collected in March 2022 from the Horticulture Research Center (HRC, Ministry of Agriculture, Giza, Egypt). One kilogram of the roots was washed and mixed in a blender, then strained. One liter of the strained juice was lyophilized, resulting in 60 g of the dry powder of beetroot juice (BRJ), which was kept in an airtight container at −20 °C for further analysis.

### 2.3. HPLC Analysis

HPLC Agilent 1200 infinity instrument (Agilent Technologies, Inc., Santa Clara, CA, USA) equipped with RP-18 column (25 × 0.5 cm, 5 µm), an auto-sampler, and a DAD detector was used for analysis. The mobile phase system comprised water with 0.1% trifluoroacetic acid (solvent A) and acetonitrile (solvent B). Gradient programming was applied starting with 90% A, then it was gradually decreased to reach 85% at 20 min. Then, the column was washed with 50% A and 50% B for 3 min. At 23 min, solvent A was restored to 90% and remained for 2 min for equilibration before the next run, with a flow rate of 1 mL/min, a 20 μL injection volume, and a column temperature of 25 °C. Absorbance recognition was conducted at 538 nm. Identification of betanin was performed by comparing their retention times with that of the standard solution of authentic betanin. The standard stock solution of betanin prepared in 50% methanol (100 mg/mL) was thus diluted to yield six concentrations (10, 20, 30, 40, 50, and 100 μg/mL). Each prepared dilution was injected three times. The mean peak area was plotted versus concentration to construct the calibration curve. Linearity was evaluated with linear regression analysis, and calculated by using the least square method.

### 2.4. Spectroscopic Properties of BRJ and Standard Betanin

BRJ and standard betanin solutions in phosphate-buffered saline (PBS) were scanned for their absorption spectra via a double-beam spectrophotometer (Rayleigh 2601, Beijing, China). The solutions (with a concentration of 2.5 mg/mL) were scanned in the visible light range (400–800 nm) using PBS as a blank.

### 2.5. Evaluation of Singlet Oxygen Generation 

The generation of singlet oxygen in aqueous irradiated solutions of BRJ and standard betanin was determined by using a reagent composed of p-nitrosodimethylaniline (RNO)/imidazole mixture as an oxygen scavenger [21]. Briefly, an aqueous solution of 5 μM of RNO and 15 mM of imidazole was mixed with 1 mg/mL of aqueous BRJ solution and with 1 mg/mL of standard betanin aqueous solution. The concentrations were adjusted till the absorption peak of RNO at 440 nm became around 0.8, and that of BRJ and betanin at 535 nm became around 0.5. Aqueous Rose Bengal solution was studied under identical conditions as a standard singlet oxygen generator. 

A total of 3 mL of each mixture was placed in a quartz cuvette and subjected to 5-min irradiation cycles using an LED source emitting light at 535 nm and 100 mW/cm^2^. The absorbance of RNO at 440 nm was recorded every 5 min for 45 min using a double-beam spectrophotometer (Rayleigh 2601, Beijing, China).

### 2.6. Molecular Dynamic Simulation Study (MD)

Using the code 2O2F [22], the 3D structures of Bcl-2 (B-cell lymphoma 2) were obtained from the protein data bank and created via UCSF Chimera. Docking calculations were done by using a molecular docking software (AutoDock Vina, San Diego, CA, USA) [23]. 

The molecular dynamic (MD) simulations for each system were performed using the PMEMD engine on the GPU, which is a component of the AMBER 18 package [24]. The CPPTRAJ module of the AMBER18 suite was utilized to analyze the trajectories after being saved every 1 ps from the MD simulations. All the visualizations were constructed by Chimaera and the Origin data analysis software [23]. 

For the calculation of the binding free energy changes (ΔG), the Poisson-Boltzmann or generalized Born and surface area continuum solvation approach (MM/PBSA and MM/GBSA) were applied [25].

The individual contribution from each residue to the overall binding free energy was determined using AMBER18’s MM/GBSA-binding free energy approach. 

Moreover, the principal component analysis (PCA) was performed to identify the conformational changes and to characterize the proteins’ atomic displacement during dynamics simulations [26]. For further assessment of Bcl-2 conformational changes after betanin engagement, dynamics cross-correlation matrices analysis (DCCM) was run on the Cα position during the simulations. 

The details of the molecular docking, molecular dynamics, thermodynamic calculations, PCA, and DCCM are provided in the Supplementary Materials.

### 2.7. Preparation of Liposomes Loaded with BRJ (Lip-BRJ) and with Standard Betanin (Lip-Bet)

Liposomes loaded with BRJ (Lip-BRJ) and with standard betanin (Lip-Bet) were prepared by reverse phase evaporation technique (REV) [27]. The liposomal membrane was formed from mixed lipids of L-α-phosphatidylcholine from soybean and cholesterol at a 10:1 *w*/*w* ratio. First, the organic phase (500 mg of L-α-phosphatidylcholine from soybean and 50 mg cholesterol dissolved in 2:1 chloroform: methanol mixture) was added to the aqueous phase (5 mL PBS containing 100 mg BRJ or standard betanin). Then, the two phases were homogenized for 10 min at 13,500 rpm using a homogenizer (Ultra-Turrax T25 laboratory emulsifier, Ika, Staufen, Germany). The evaporation process was in a rotary evaporator (Heidolph Elektro GmbH & Co. KG, Schwabach, Germany) at 200 rpm under vacuum. The temperature was adjusted to be slightly higher than the phase transition temperature of the used lipid [17]. The evaporation was continued until a viscous gel was formed. The liposomal vesicles were formed upon hydration of the viscous gel by PBS, and the solution was returned to the rotary evaporator to remove any remaining organic solvent. The final volume was adjusted to 20 mL by PBS.

To reduce the size, the liposomal suspensions were sonicated for 10 min. Finally, the prepared liposomal suspensions were stored at 4 °C for further investigations. 

### 2.8. Characterization of the Prepared Liposomes

For measurement of the encapsulation efficiency (EE) and the loading capacity (LC), the unencapsulated BRJ and standard betanin were separated from the liposomal suspensions using centrifugation (Centrikon T-42 K, Kontron Instruments, Milan, Italy) for 30 min at 10,000 rpm and 6 °C. The precipitated liposomes were dissolved in ethanol to dissolve lipids and diluted by PBS (pH = 7.4). The absorbance of BRJ and standard betanin in the liposomal suspensions was measured spectrophotometrically at 535 nm, as mentioned above. Then, BRJ and betanin concentrations were calculated from a standard calibration curve previously constructed in an (ethanol: PBS) mixture. Measurements were done in triplicate and results were represented as means ± standard deviations. Finally, EE and LC were calculated as follows: 

EE = (calculated BRJ or betanin amount in the liposomes/initial BRJ or betanin amount used in the preparation) × 100.

LC = (calculated BRJ or betanin amount in the liposomes/initial lipid amount used in the preparation) × 100.

The particle size analyzer (Nano-ZS, Malvern Instruments Ltd., Worcestershire, UK) was used to measure the average liposomal particle size and zeta potential. Liposomal suspensions were diluted, in a 1:100 ratio, with distilled water and sonicated for 10–20 min just before assessment. Measurements were done in triplicate, and results were represented as means ± standard deviations.

The liposome’s shape and structure were examined by a high-resolution transmission electron microscope (HR-TEM, JEM-2100 plus, Tokyo, Japan). Before being examined, the unstained liposomal suspension was diluted with distilled water and applied on a copper grid. 

The release of betanin from the prepared liposomes was investigated as previously reported [27] with minor modifications. In a 150 mL glass beaker, 1 mL of the BRJ liposomes, betanin liposomes, and aqueous betanin solution (equivalent to 5 mg/mL) were put into three separate dialysis bags (molecular weight cut-offs of 12,000–14,000 Da), each bag (2 cm^2^ area) was submerged in 100 mL PBS (pH 7.4) at 37 °C while continuously stirring at 100 rpm. To maintain sink conditions, the volume of the release media was adjusted to be ten times greater than required to attain the saturation point of betanin. In the predetermined time intervals, 2 mL of the accepting solution was removed and replaced by an equal volume of fresh PBS. The released BRJ and betanin concentrations were measured by spectrophotometry at 535 nm. This procedure was carried out three times, and then the mean cumulative release of BRJ and betanin was computed and displayed as a function of time. StatistiXL 2.0 for MS Excel software was used to model the obtained data to zero order, first order, and Higuchi’s diffusion control models by employing the coefficient of variation for data analysis. The release kinetics were assessed with the least coefficient of variation and the greatest linear correlation value (R^2^). 

### 2.9. In Vitro Assessment of Photodynamic Activity 

#### 2.9.1. Cell Lines and Cell Culture

The photodynamic activity of BRJ, standard betanin, Lip-BRJ, and Lip-Bet was assessed using the determination of the dark and photo-cytotoxicity of the tested samples on two cell lines: a normal cell line (HEK 293; Human embryonic kidney 293 cells) and a cancerous cell line (A549 human lung carcinoma cells). Both were purchased from America Type Culture Collection (ATCC) and were propagated in DMEM culture media with 10% FBS and 1% penicillin-streptomycin antibiotic. The cells were incubated in a humidified environment with 5% CO_2_ at 37 °C and routinely inspected for contamination.

#### 2.9.2. Dark Cytotoxicity

In 96-well plates, 100 μL cell suspension aliquots (about 4000 cells per well) were seeded and incubated in DMEM media for 24 h till complete adherence. Then, the media were replaced by new media containing serial concentrations of the tested samples (from 100 to 2000 μg/mL) and incubated in the dark for a further 24 h. Some cells were incubated with empty liposomal suspension with concentrations equivalent to the concentration of the tested samples (the highest concentration used of empty liposomal suspension was 400 μL/mL). Control cells were incubated with fresh media only without any samples. Afterward, cell viability was evaluated by applying the MTT colorimetric assay. Briefly, the preceding media was replaced by a new one containing 10% MTT, and the cells were incubated for 4 h to permit the MTT reduction by the viable cells’ mitochondrial enzymes. Then, the media were withdrawn, the cells were rinsed with PBS, and 200 μL of DMSO was added to each well to dissolve the produced formazan crystals. Each experiment was repeated for at least three times and the results were interpreted as means ± SEs. The absorbance was measured with an ELISA microplate reader (Biotek ELx800, Temecula, CA, USA) at 570 nm. Finally, the cell viability of the treated cells was determined as follows: 

The % cell viability = absorbance recorded for the treated cells/Absorbance recorded for the control cells × 100. 

#### 2.9.3. Photocytotoxicity 

After 24 h of incubation with samples, the cells were washed with PBS and exposed to light radiation for 20 min. The light was delivered through an LED light source (locally manufactured at the National Institute of Laser Enhanced Sciences, Cairo University, Egypt) at 535 nm and 50 mW/cm^2^. The power was measured by a power meter (Edmund Optics Inc., Barrington, NJ, USA).

After radiation, the cells were incubated in full media for a further 24 h, and this was followed by performing the MTT assay as described above.

##### Statistical Analysis 

The data were statistically analyzed using a two-way analysis of variance (ANOVA) followed by Bonferroni multiple comparison tests using GraphPad Prism software v 5.01 (Graphpad, San Diego, CA, USA). Significance was considered when the *p*-value was smaller than 0.05 (*p* < 0.05). Results were interpreted as means ± standard errors (SEs).

## 3. Results

### 3.1. HPLC of Betanin in Beetroot Extract

**Figure 1:**
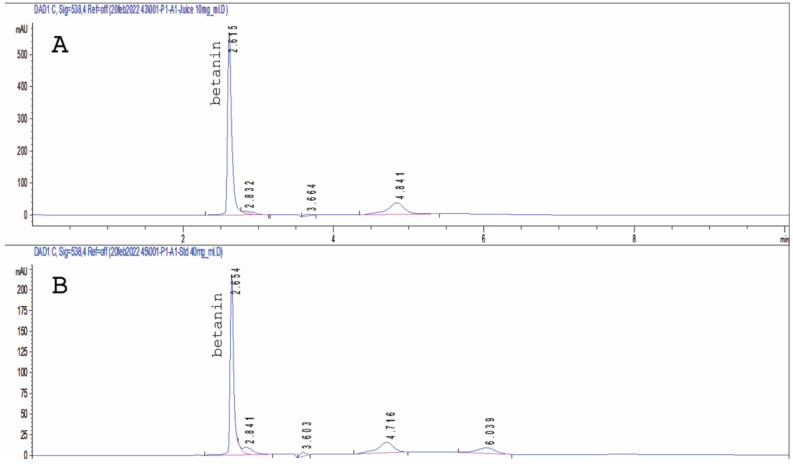
HPLC chromatogram of beetroot juice (**A**) and standard betanin (**B**) using RP-18 column at λmax 538 nm.

The HPLC chromatogram of beetroot juice allowed the identification of betanin at 538 nm as compared to the standard. Betanin was quantified (Figure 1) in the beetroot juice sample by calculating its amount using an established calibration curve. The amount of betanin calculated was 0.6 mg/mg dry juice extract.

The standard calibration curve has a correlation coefficient (R^2^) of 0.99988, indicating the linearity of betanin’s peak area in the 10–100 mg/mL range. Quantification of betanin in beetroot juice was achieved from the least square regression equation of the calibration curve of the standard.

### 3.2. Spectroscopic Properties of BRJ and Betanin

**Figure 2:**
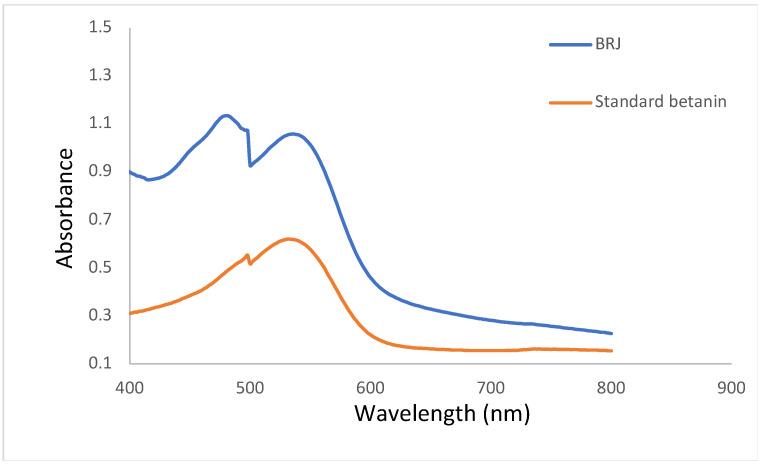
The absorption spectra of beetroot juice (BRJ) and standard betanin.

Figure 2 reveals strong absorption of BRJ and betanin solutions in the visible region with a maximum absorption peak at 535 nm. Moreover, the spectrum of BRJ exhibited another distinct peak at 480 nm due to the yellow dye betaxanthin. The presence of these two peaks (535 nm and 482 nm) was formerly reported [5,7,28]. 

### 3.3. Singlet Oxygen Generation

**Figure 3:**
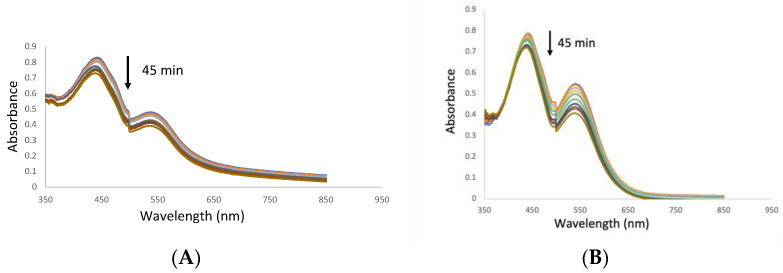
The decrease of RNO absorbance at 440 nm after irradiation in the aqueous solution of the beetroot juice (**A**) and the standard betanin (**B**).

The RNO/imidazole technique was used to monitor singlet oxygen formation in aqueous BRJ and standard betanin solutions. Monitoring the absorbance of RNO at 440 nm for 45 min of irradiation (divided into cycles of 5 min of irradiation) revealed a 12% decrease for BRJ (Figure 3A) and a 9% decrease for standard betanin (Figure 3B). The absorption peak at 535 nm corresponding to betanin was also decreased upon increasing the radiation period, while there was no significant change in the absorbance of the RNO/imidazole solution kept in the dark for about 1 h. On the other hand, the Rose Bengal solution caused a 94% decrease in RNO absorbance after 35 min of radiation. 

### 3.4. Molecular Docking and Molecular Dynamic Simulation 

#### 3.4.1. Molecular Dynamic and System Stability 

**Figure 4:**
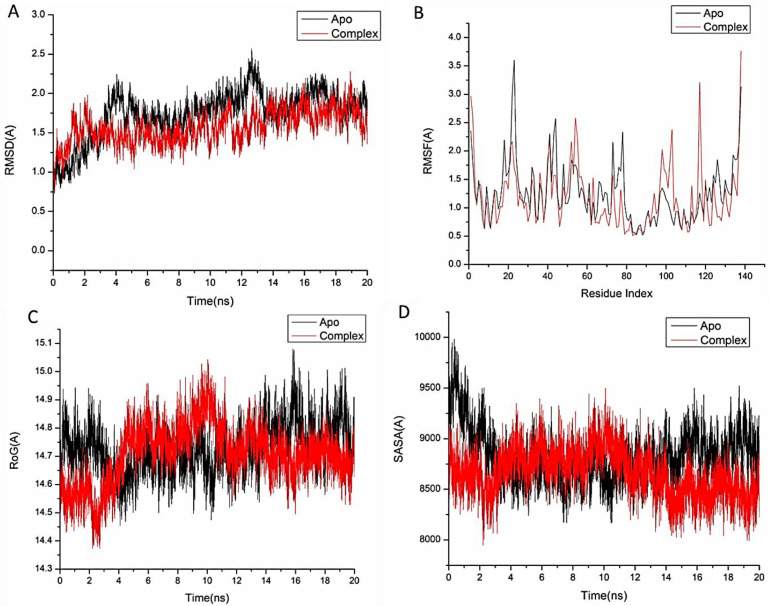
(**A**) The root-mean-square deviation (RMSD) of Cα atoms of the protein backbone atoms (black) and Bcl2 receptor with betanin (red) during the entire 20 ns. (**B**) The root-mean-square fluctuation (RMSF) of each residue of the protein backbone Cα atoms of protein residues (black) and Bcl-2 receptor with betanin (red). (**C**) ROG of Cα atoms of protein residues (black) and Bcl-2 receptor with betanin (red). (**D**) solvent-accessible surface area (SASA) of the C α of the backbone atoms relative (black) and Bcl-2 receptor with betanin (red).

To anticipate the behavior and stability of betanin interaction following the binding to the Bcl-2 active site, a molecular dynamic simulation model was conducted [29,30]. RMSD (root-mean-square deviation) was used for the evaluation of the system stability throughout 20 ns simulations. The entire frames of the systems had average RMSD values of 1.73 ± 0.29 É and 1.57 ± 0.20 Å for Bcl2-apo and Bcl2-betanin complexes, respectively (Figure 4A).

In addition, the RMSF (root-mean-square fluctuation) technique was employed to examine protein residue variation and the influence of betanin-target binding over 20 ns simulations. Bcl2-apo and Bcl2-betanin complex average RMSF values were 1.26 ± 0.52 Å and 1.19 ± 0.56 Å, respectively (Figure 4B). These results showed that, in comparison to the other systems, the betanin-protein complex system exhibited minimum protein residue variations. 

The ROG (radius of gyration) assessed both the system stability and its compactness during binding to the ligand [23]. As shown in Figure 4C, the Bcl2-apo and Bcl2-betanin complexes exhibited average ROG values of 14.73 ± 0.08 Å and 14.70 ± 0.10 Å, respectively. The discovered behavior indicates that the betanin molecule has a very stiff structure when it comes to the Bcl2 receptor. 

The solvent-accessible surface area (SASA) of the protein was evaluated to investigate the compactness of the Bcl-2 hydrophobic core. The Bcl2-apo and Bcl2-betanin complex average SASA values were 8820.01 Å and 8661.18 Å, respectively (Figure 4D). The combined results of the SASA, RMSD, RMSF, and ROG values, verified the stability of the betanin inside the catalytic domain of the Bcl2-receptor binding site. 

#### 3.4.2. The Binding Free Energy Thermodynamic Calculations 

The change in binding free energies was measured for the betanin-Bcl2 complex, as illustrated in Table 1. Except for ΔGsolv, all the calculated energy components exhibited high negative values, suggesting favorable interactions. All inhibitory activities get values as high as −195.28 kcal/mol in the gas phase.

#### 3.4.3. Identification of the Residues Involved in Betanin Binding to Bcl2 Receptors and Interaction Network Profiles

**Figure 5:**
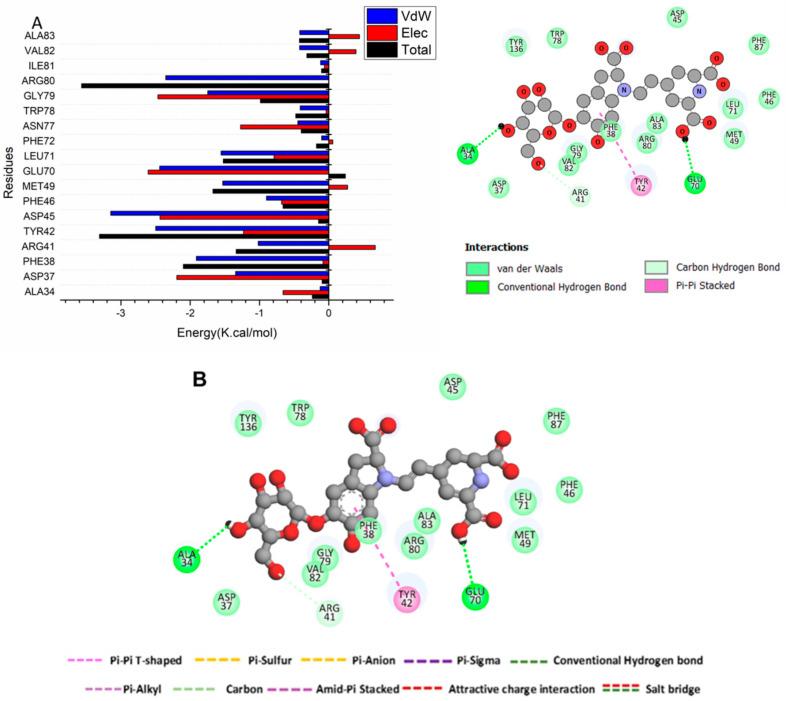
(**A**) Per-residue decomposition analysis illustrating individual energy contributions to the binding of betanin at the hydrophobic grooves of Bcl-2 receptor. (**B**) The interaction residue of betanin into the Bcl-2 receptor catalytic site.

The total energy for betanin-Bcl2 binding was subsequently analyzed to identify the participation of different site residues. The identified crucial residues engaged in the inhibition of the ATP binding site of the Bcl-2 receptor (as illustrated in Figure 5) were Asp37 (−1.346 kcal/mol), Phe38 (−1.912 kcal/mol), Arg41 (−1.018 kcal/mol), Arg47 (−1.213 kcal/mol), Phe65 (−2.932 kcal/mol), Arg69 (−0.605 kcal/mol), Tyr42 (−2.499 kcal/mol), Asp45 (−3.148 kcal/mol), Met49 (−1.53 kcal/mol), Glu70 (−2.44 kcal/mol), Leu71 (−1.554 kcal/mol), Asn77 (−0.447 kcal/mol), Trp78 (−0.412 kcal/mol), Gly79 (−1.746 kcal/mol), Arg80 (−2.345 kcal/mol), Val82 (−0.421 kcal/mol), and mAla83 (−0.419 kcal/mol).

Figure 5B shows that the chemical structure of betanin fits into the catalytic active site of Bcl-2. Betanin has been discovered to form a stable hydrogen bond contact between Glu 70 and Ala 34. Furthermore, a Pi-Pi stack has developed between Tyr 42 and betanin.

#### 3.4.4. Principal Component Analysis (PCA)

**Figure 6:**
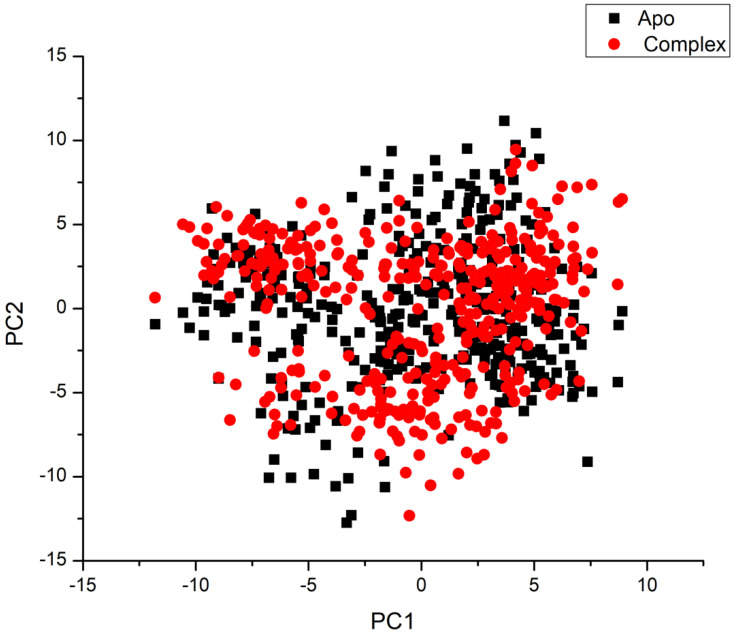
The first two principal components (PC1 and PC2) plotted in the conformational space with APO (black) and the betanin complex (red) resulted in a PCA projection of the mobility of Ca atoms. A covariance matrix is represented as PC1 and PC2, respectively, following the removal of eigenvectors (rotational motions). Similar structural conformations overlap on the graph, and each point between the single-directional motions indicates a distinct conformation during the simulation.

Figure 6 illustrates clear and complex waves of conformation along the two major components in significant subspace. The betanin complex and apo-protein systems all demonstrated a noticeable difference in motion. As a result, the computed eigenvector from the 20 ns MD trajectories for the systems is very variable, demonstrating the protein motion variation among the systems.

Compared to the betanin-complex system, the Bcl2-apo system exhibits greater atomic fluctuations, suggesting that the ligand’s adherence to the active site produces conformational dynamics that are observed as a wave of motion. The betanin-complex system, on the other hand, is more densely packed than the other system. When betanin binds to the protein, conformational flexibility is decreased, boosting the binding of betanin to the active site.

**Figure 7:**
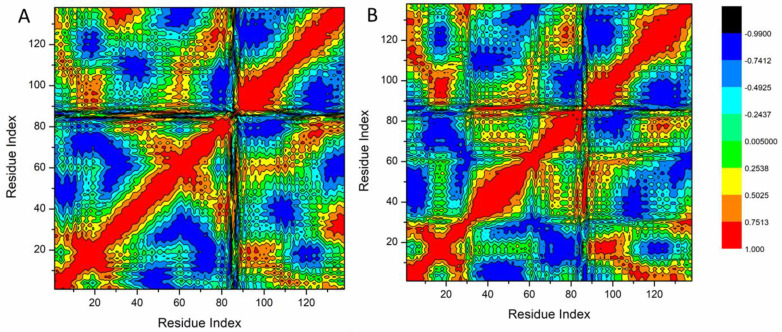
Dynamic cross-correlation matrix analyses for Apo-AR (**A**) and betanin binding to Bcl-2 proteins (**B**). Numbers closer to 1 indicate a high correlation, while those closer to −1 indicate anticorrelation between pairs of residues.

The DCCM study revealed that certain residues exhibit extremely positive-correlated motions (yellow-red regions), whereas certain residues exhibit highly negative-correlated motions (blue-black regions; Figure 7).

The systems under investigation that were assessed displayed the overall correlated motions of residues in contrast to the anti-correlated motions. An examination of the DCCM indicates that betanin binding to the Bcl-2 proteins resulted in conformational alterations in the protein structure, which are reflected in differences in the corresponding movements. There are associated portions in the betanin binding to Bcl-2 proteins, as shown in Figure 7. The Bcl-2 protein residues 40–6- and 120–138 contain a substantially correlated area. Most hydrophobic active site residues are accepted by these highly dynamic regions of the receptor.

### 3.5. Preparation and Characterization of Liposomes

BRJ and standard betanin were successfully encapsulated in liposomes (Lip-BRJ and Lip-Bet, respectively) using the reverse phase evaporation method (REV). The determined EE, LC, mean particle size, and zeta potential are summarized in Table 2.

**Figure 8:**
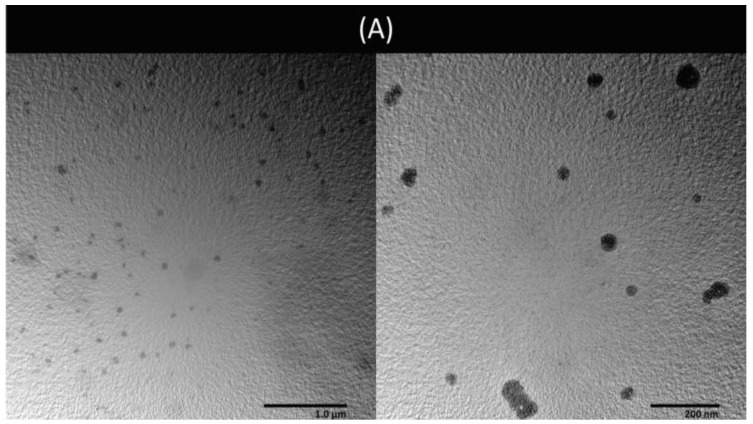

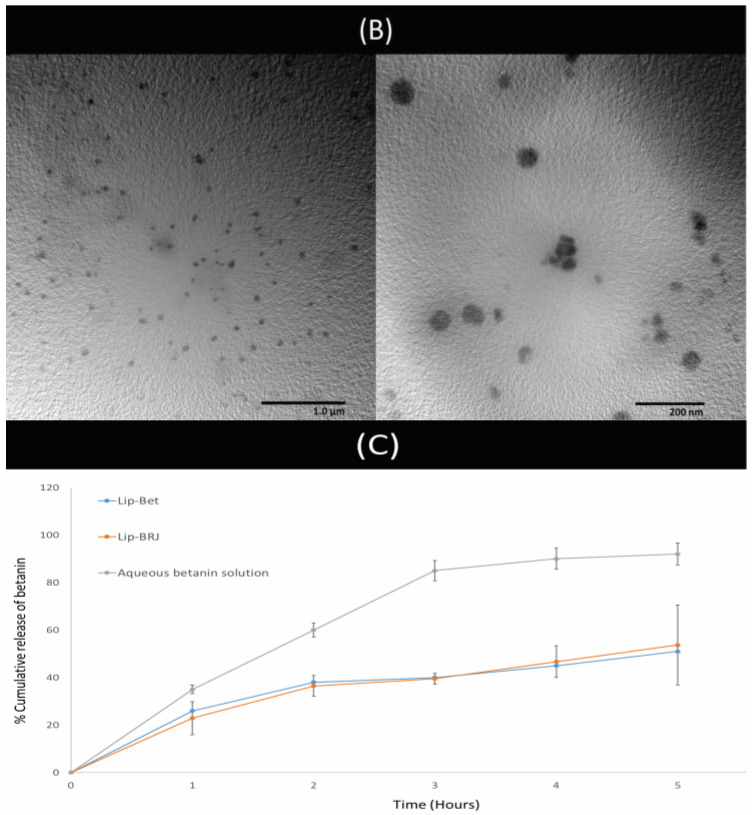
(**A**) TEM photo of liposomal beetroot juice, (**B**) TEM photo of liposomal betanin, (**C**) cumulative release of the betanin from the liposomal beetroot juice, liposomal betanin, and aqueous betanin solution.

As hydrophilic compounds, BRJ and betanin were encapsulated in the inner aqueous core of the spherical liposomal vesicles, as revealed from the TEM photos (Figure 8A,B). 

Betanin was rapidly released from the aqueous betanin solution (92 ± 5% was released after 5 h). On the other hand, betanin was released from the liposomal membrane in a controlled manner (Figure 8C). About 54 ± 16% and 51 ± 10% of betanin was released from Lip-BRJ and Lip-Bet after 5 h, respectively. The release was best fitted with Higuchi’s diffusion (R^2^ = 0.97), suggesting that the mechanism is the diffusion through the phospholipid bilayer of the liposomal membranes [27]. 

### 3.6. In Vitro Photodynamic Activity 

**Figure 9:**
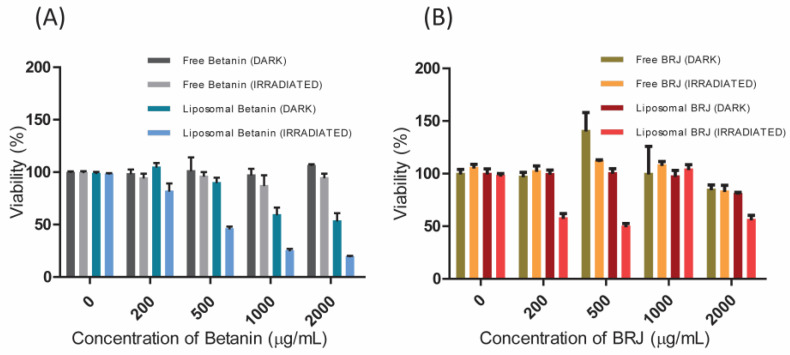
The dark and photocytotoxicity on the normal (HEK 293) cell line exerted by (**A**) free and liposomal betanin and (**B**) free and liposomal BRJ (beetroot juice). Each experiment was repeated three times. Data were analyzed using Graphpad Prism v. 5.01 (Graphpad, San Diego, CA, USA), and the results were interpreted as means ± SEs.

As illustrated in Figure 9A, the standard betanin was safe on the normal cell line at all tested concentrations with no significant difference between dark and photo-cytotoxicity (*p*-value > 0.05). The encapsulation of betanin into liposomes enhanced both dark and photo-cytotoxicity in a concentration-dependent manner. Irradiation has significantly (*p*-value < 0.01) enhanced the cytotoxicity of Lip-Bet (only 20% cell viability was recorded at 2000 µg/mL betanin). 

Figure 9B showed that BRJ exerted non-significant dark cytotoxicity on the normal cells at the tested concentrations, indicating its high safety. In the case of Lip-BRJ, the light irradiation significantly enhanced the cytotoxicity on normal cells (*p*-value < 0.05) at low concentrations (200 and 500 µg/mL). At higher concentrations, light did not improve the cytotoxicity of Lip-BRJ.

**Figure 10:**
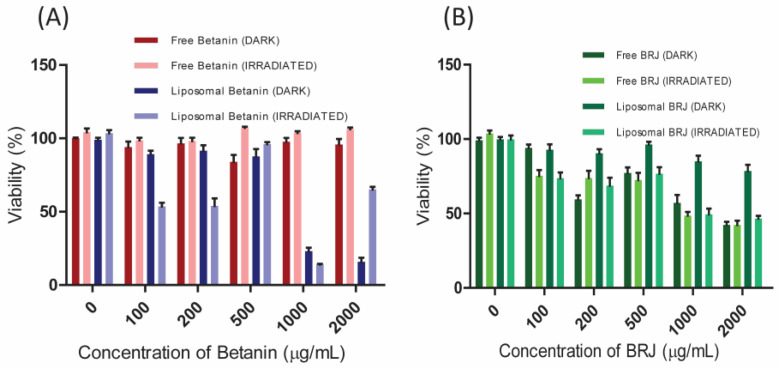
The dark and photo-cytotoxicity on the human lung cancer (A549) cell line exerted by (**A**) free and liposomal betanin and (**B**) free and liposomal BRJ (beetroot juice). Each experiment was repeated three times. Data were analyzed using Graphpad Prism v. 5.01 (Graphpad, San Diego, CA, USA), and the results were interpreted as means ± SEs.

The results of the cytotoxicity experiment on the lung cancer cells revealed that the standard betanin (free betanin) exhibited negligible dark and photo-cytotoxicity (Figure 10A). 

Lip-Bet exerted significant dark cytotoxicity on lung cancer cells at high concentrations (cell viability of 23% and 15% were obtained at 1000 and 2000 µg/mL, respectively). 

Light irradiation significantly enhanced the cytotoxicity of Lip-Bet at low concentrations (100 and 200 µg/mL). However, light did not impart any significant enhancement at higher concentrations.

On the other hand, BRJ exerted pronounced dark cytotoxicity on lung cancer cells (Figure 10B) at high concentrations (at 2000 µg/mL of BRJ), and the cells exhibited viability of 42%), indicating that the cancer cells are more sensitive to the BRJ. In the case of Lip-BRJ, the light irradiation significantly enhanced the cytotoxicity on the lung cancer cells at all concentrations (Figure 10B). 

Empty liposomes (with zero betanin and BRJ concentrations) did not impart significant dark or photo-toxicity to the used cell lines. They resulted in cell viability of about 100%, as was obtained in the case of untreated control cells (Figure 9 and Figure 10). 

## 4. Discussion

Using plant extracts as photosensitizers in PDT is a significant green approach for achieving sustainability. In the present study, we attempted to investigate a new use for the well-known beetroot extract juice (BRJ) as a photosensitizer in the PDT of cancer. Betanin is the main active phytoconstituent in BRJ, as revealed from the HPLC chromatogram (Figure 1). Moreover, it is the dye that is mainly responsible for the light absorption properties of BRJ.

Based on the visible spectroscopy results, we can deduce that beetroot extract and standard betanin can be activated by light radiation at 535 nm. Upon transferring this energy to the available oxygen, singlet oxygen and reactive oxygen species can be formed, destroying many cellular components [5]. The generation of singlet oxygen was further verified by the RNO/imidazole technique. It depends on the presence of an oxygen quencher (imidazole) which quenches the oxygen and forms an intermediate compound leading to the bleaching of the oxygen sensor (RNO) [21]. The decrease in the RNO absorbance (at 440 nm) upon increasing the irradiation period (Figure 3A,B) reflects the formation of singlet oxygen. The singlet oxygen is formed at low levels compared to a well-known singlet oxygen generator such as Rose Bengal. Nevertheless, the singlet oxygen production can verify that standard betanin and BRJ are promising photosensitizers. 

To go deeper into proposing a possible mechanism of betanin’s PDT and anticancer effects, we carried out investigations using molecular dynamic modeling. 

Molecular dynamic is a computational technology that plays a crucial role in drug research to identify compounds with therapeutic potential. Docking experiments are conducted to determine the optimal orientation and molecular interactions between natural chemicals and specific proteins [31]. 

Molecular dynamic investigation was previously conducted to test the betanin binding affinity to Sentrin-Specific Protease1 [32]. In the current work, the selectivity mechanism of betanin interaction with Bcl-2 proteins was examined using binding free energy analysis and comparative MD simulation. By applying the MM/GBSA method, the binding of betanin to these protein targets was evaluated. The results revealed favorable interactions with ΔG values of −40.94 kcal/mol. The electrostatic energy component is mostly responsible for this effect. Moreover, the key amino residues in Bcl-2 receptor inhibition were determined. The high affinity of betanin to bind to Bcl2- proteins was further approved by the results of PCA and DCCM analysis. Our findings are crucial for comprehending the molecular underpinnings of betanin Bcl-2 receptor action and designing more effective selective inhibitors.

Before proceeding to the in vitro investigation of their photodynamic activity, BRJ and standard betanin were encapsulated in liposomes. Liposomes were prepared with the REV technique using L-α-phosphatidylcholine from soybean, which is a cost-effective, natural lipid that was reported to produce physically stable liposomes. Cholesterol was added as it enhances the liposomal membrane stability by decreasing the hydrocarbon chain mobility, hence boosting the retention of the hydrophilic extract in the liposomal aqueous core with minimal leakage [33]. Many factors should be considered in preparing liposomes with the REV method, such as the drug/lipid ratio, organic phase/aqueous phase ratio, and power of homogenization [34].

The EE and LC are dependent on several factors, such as the drug hydrophilicity, the component of the lipid membrane, and the preparation method [13]. In the ordinary thin film hydration method, low encapsulation efficiency is usually obtained for the hydrophilic compounds. On the contrary, in the REV technique, the lipid phase and the aqueous phase form an oil-in-water emulsion, which is then diluted with the aqueous phase. Consequently, the developed liposomes have high aqueous content, which enhances the encapsulation of hydrophilic drugs [35,36]. Attaining higher encapsulation is beneficial as it reduces the drug dose and, consequently, reduces the adverse effects. 

The size of Lip-BRJ liposomes was higher than Lip-Bet, which may be due to the large molecules for different components of the extracted juice. The particle size measured by TEM is smaller than the apparent size measured by the zeta sizer. The particle size analyzer using dynamic light scattering usually measures the hydrodynamic size of the particles in solution, so the size is usually shifted to larger values. In contrast, during the TEM imaging process, liposomes are subjected to dehydration and contraction, which is reflected in more realistic values [37,38]. 

The prepared liposomes exhibited high negative zeta potential (Table 2), indicating good dispersibility, low tendency of agglomeration in aqueous media, and high colloidal stability [15]. 

The in vitro photodynamic activity of BRJ, betanin, and their liposomal formulations were tested on two cell lines. HEK 293 cell line was used as a model of normal cells to investigate the safety of the tested samples. The A549 cell line, representing cancer cells, was selected to assess the vitro photodynamic activity of the tested samples. 

The results showed negligible cytotoxicity of empty liposomes, which may be attributed to the biocompatibility of the used lipid (L-α-phosphatidylcholine from soybean) [34]. 

Collectively, the results of the cytotoxicity experiment proved the positive effect of light irradiation on the anticancer activity of both betanin and BRJ, especially at low concentrations. Moreover, results revealed that the photo-cytotoxicity improved after liposomal encapsulation, suggesting the efficiency of liposomal beetroot extract as a photosensitizer in PDT of cancer. The hydrophilic nature of beetroot extract resulted in poor cellular uptake, which can be boosted by encapsulation of the beetroot extract in liposomes, thus increasing the photo-cytotoxicity [4]. When comparing the cytotoxicity results on normal to cancer cells, we can deduce that the impact of liposomes on increasing the cytotoxicity after light irradiation was more significant in the case of cancer cells. 

Additionally, results revealed that the impact of light on the cytotoxicity was sometimes paradoxically abolished at higher concentrations. This may be due to the possible interference between the absorption peak of betanin (or BRJ) at 535 nm and that of formazan crystals at 570 nm when carrying out the MTT assay, giving rise to a false high absorbance [39]. However, the results are reliable at low concentrations, substantiating the potency of BRJ and betanin liposomes as natural photosensitizers.

Beetroot juice has been extensively explored for several therapeutic and biological effects. It has been reported to possess potential antioxidant, anti-inflammatory, anticancer, antidiabetic, and neuroprotective properties [8,10,12,14,40]. The safety of the beetroot was reported by most of these studies. 

In concurrence, the anticancer activity of betanin was previously reported against several cancer types, mainly owing to its potential antioxidant properties. Zhang et al. proved that oral administration of betanin was effective against lung cancer in mice [41]. Likewise, it was reported that 200 µg/mL betanin extracted from beetroot extract caused 49% cell inhibition [42]. Similarly, the combination of betanin and doxorubicin imparted synergistic cytotoxicity on prostate, breast, and pancreatic cancer cell lines [43]. On the other hand, few reports are available about the anticancer activity of the whole beetroot extract. Romero et al. addressed the anticancer effectiveness of the beetroot and beet leaves extracts on cervical cancer and revealed that beetroot extract was a more efficient anticancer agent than its isolated compounds against Hela cells as it caused apoptosis, suppressed viability, and arrested the cell cycle [44]. In the same context, betanin-enriched beetroot extract suppressed the proliferation of MCF-7 cells but had no discernible effect on normal cells [40]. On the other hand, the anticancer effects of beetroot on the colorectal, ductal breast, and lung cancer cell lines were pronounced only after conjugation to chitosan nanoparticles [45]. 

Regarding the efficacy of beetroot extract and betanin as photosensitizers, Mittal et al. investigated the efficacy of beetroot extract as a photosensitizer in antifungal-PDT to eradicate *Candida albicans* [5]. Apart from medical applications, some studies investigated beetroot extract and betanin as photosensitizers in solar cells [7,28,46]. However, to our knowledge, no reports about the efficacy of beetroot extract or betanin, especially loaded in liposomes, as photosensitizers in PDT of cancer, and the related data were depicted. 

Employing liposomes for in vivo applications adds value as it prolongs the circulation time, shields the drug from oxidation and hydrolysis, and can passively target the photosensitizer to tumor tissues [1]. Our in vitro study results may represent a basis for further in vivo and clinical studies.

Several previous studies have addressed the efficiency of natural photosensitizers in PDT [2,3,47,48]. In this work, we focused on the photodynamic activity of beetroot juice, which is due to its ability to generate singlet oxygen upon excitation by light at 535 nm. However, this excitation wavelength (535 nm) is shorter than the optical window (600–800 nm), resulting in a limited depth of penetration. Nevertheless, several previous studies have addressed the use of photosensitizers like Rose Bengal and Eosin Yellow, which absorb light at the same wavelength (500–530), in various topical PDT applications [49,50,51,52]. Consequently, beetroot juice can be introduced as a promising natural cheap photosensitizer for PDT of superficial tissues. 

## 5. Conclusions

Searching for a safe, economical, and easily available plant-derived photosensitizer was the main goal of this study. In this way, we investigated the potential of liposomal beetroot juice and its active constituent betanin as green photosensitizers in photodynamic therapy. The molecular dynamic study deduced that betanin has a high affinity for binding to Bcl-2 proteins. These results suggest that betanin may act as a potent Bcl-2 inhibitor, accounting for its anticancer and photodynamic activities. Moreover, BRJ and betanin exhibited promising photodynamic activity on cancer cell lines, which was enhanced after being encapsulated in liposomes. The results of our in vitro study may represent a basis for further in vivo and clinical investigations.

## Figures and Tables

**Table 1:** The estimated energy binding for the betanin compound versus the Bcl2 receptor.

Energy Components (kcal/mol)					
Protein Complex	ΔE_vdW_	ΔE_elec_	ΔG_gas_	ΔG_solv_	ΔG_bind_
Betanin-Bcl2	−42.42 ± 0.36	−152.86 ± 1.63	−195.28 ± 1.61	154.34 ± 1.37	−40.94 ± 0.42

∆E_vdW_ = van der Waals energy; ∆E_ele_ = electrostatic energy; ΔG_gas_ = the change in the Gibbs free energy of the gas; ∆Gs_olv_ = solvation free energy; ∆G_bind_ = calculated total binding free energy.

**Table 2:** Encapsulation efficiency, loading capacity, particle size, zeta potential, and PDI of the prepared liposomes.

Liposomes	Encapsulation Efficiency %	Loading Capacity %	Particle Size (nm)	Zeta Potential (mV)	PDI
Empty Liposomes	-	-	210 ± 20	−19 ± 6.0	0.3
Lip-BRJ	61.9 ± 5.3	12 ± 4.0	441.5 ± 160	−43.7 ± 7.5	0.4
Lip-Bet	65.2 ± 3.0	14 ± 3.0	233.0 ± 60	−23.0 ± 8.0	0.4

Data are expressed as mean ± S.D.

## Data Availability

The datasets generated during and/or analyzed during the current study are available from the corresponding author upon reasonable request.

## Supplementary Materials

The following supporting information can be downloaded at: https://www.mdpi.com/article/10.3390/pharmaceutics16081038/s1. Supplementary Materials: Details of the molecular docking, molecular dynamics, thermodynamic calculations, PCA, and DCCM.
