# Supplementary material for: Nano-Liposomal Beetroot Phyto-Pigment in Photodynamic Therapy as a Prospective Green Approach for Cancer Management: In Vitro Evaluation and Molecular Dynamic Simulation
Source: Pharmaceutics. 2024 Aug 3;16(8):1038. doi: 10.3390/pharmaceutics16081038 (PMC11360503; doi:10.3390/pharmaceutics16081038)
Supplement: Supplementary file 1 [file pharmaceutics-16-01038-s001.zip › pharmaceutics-3072733-supplementary.pdf]

Methodology of Molecular Dynamic Study

S1. System preparation and molecular docking

Using the code 2O2F (Bruncko et al., 2007) the 3D structures of B-cell lymphoma 2 (Bcl-2) were obtained from the protein data bank and created via UCSF Chimera. The pH was adjusted to 7.5 with PROPKA. Using ChemBioDraw Ultra 12.1, the synthetic 2D structure was shown. The Avogadro software employed the steepest descent approach and the MMFF94 force field to optimize the 2D structure for energy minimization. UCSF chimaera was utilized to eliminate hydrogen atoms before docking.

Docking calculations were done by applying AutoDock Vina, and Gasteiger partial charges were assigned during the docking process. The AutoDock atom types were delineated using the AutoDock graphical user interface provided by MGL tools. The AutoDock Vina grid center coordinates for x, y, and z are 1.2653 Å, 4.56347 Å, and 4.75153 Å. The search space's dimensions were 20 Å x 20 Å x 20 Å, with an exhaustiveness of 8. Docked conformations were generated according to their docking energy using the Lamarckian genetic method in descending order.

S2. Molecular dynamic (MD) simulations

The MD simulations for each system were performed using the PMEMD engine on the GPU, which is a component of the AMBER 18 package (Lee et al., 2018).

The partial atomic charge was calculated using the General Amber Force Field (GAFF) method created by ANTECHAMBER. The AMBER 18 package's Leap module solved each system within an orthorhombic box containing TIP3P water molecules within 10 Å of each edge. Na+ and Cl^- counter ions were introduced by the Leap module for system neutralization. An initial minimization (2000-step) using a 500 kcal/mol imposed restraint potential and a full minimization (1000-step) using the conjugate gradient approach in the absence of constraints were applied to each system.

To guarantee the homogeneity of systems during the MD simulation, each system was heated incrementally over 500 ps, from 0K to 300K. The solutes in the system experienced a collision frequency of 1 ps and a potential harmonic limitation of 10 kcal/mol. Every system was then heated to a constant temperature of 300K and allowed to equilibrate for 500ps. The number of atoms and pressure in each system were kept constant to replicate an isobaric-isothermal ensemble (NPT). The system's pressure was then kept at 1 bar via a Berendsen barostat.

Every system was MD simulated for 20 ns. In every simulation, the hydrogen bond atoms were constrained using the SHAKE approach. Every simulation integrated an SPFP precision model and used a 2fs step size. Simulations were conducted using an isobaric-isothermal ensemble (NPT) with randomized seeding, constant pressure of 1 bar, pressure-coupling constant of 2ps, temperature of 300K, and Langevin thermostat with collision frequency of 1ps.

The CPPTRAJ module of the AMBER18 suite was used to analyze the trajectories after they were saved every 1 ps from the MD simulations. All the graphs and visualizations were made using Chimaera and the Origin data analysis software.

S3. Thermodynamic calculations (Binding free energy calculations)

The protein-ligand binding affinities were determined using the Poisson-Boltzmann or generalized Born and surface area continuum solvation approach (MM/PBSA and MM/GBSA (Tuccinardi, 2021).

The change in binding free energy (ΔG) for each molecular species, including complex, ligand, and receptor can be represented by the equations (1) and (2) :

$$\Delta G_{bind} = G_{complex} - G_{receptor} - G_{ligand} \quad \text{(S1)}$$

$$\Delta G_{bind} = E_{gas} + G_{sol} - TS \quad \text{(S2)}$$

Where Egas and Gsol represent the gas-phase energy and the solution-free energy, respectively. The total entropy of the solute and temperature are represented by items S and T, respectively.

The FF14SB force field were used to directly assess the Egas and represented by equation (S3):

$$E_{gas} = E_{int} + E_{vdw} + E_{ele} \quad \text{(S3)}$$

Where Eint, Evdw, and Eele are the internal energy, van der Waals energy, and Coulomb energy, respectively. The energy of the polar states (GGB) and non-polar states (G) was used to calculate the Gsol, as represented in equation 4:

$$G_{sol} = G_{GB} + G_{SA} \quad \text{(S4)}$$

Using a water probe radius of 1.4 Å, the non-polar solvation-free energy (GSA) was calculated from the Solvent Accessible Surface Area (SASA) as in equation (S5):

$$G_{SA} = \gamma SASA \quad (S5).$$

The individual contribution from each residue to the overall binding free energy was determined using Amber18's MM/GBSA-binding free energy approach.

S4. Principal Component Analysis (PCA) and dynamics cross-correlation matrices (DCCM) analysis

PCA is a multivariate statistical method that screens observed motions from the biggest to smallest spatial scale. By identifying several conformational modes of the protein complex during dynamics simulations, the PCA technique can characterize the proteins' atomic displacement and conformational changes. The biological system's eigenvalues and eigenvectors, or direction and extent of motion, can also be found using PCA (Ylilauri and Pentikäinen, 2013). Here, the CPPTRAJ module in Amber18 was used to remove the ions and solvent molecules from 20 ns of MD trajectories (Ylilauri and Pentikäinen, 2013). This was completed before PCA's MD trajectory processing. PCA was applied, using in-house scripts, on Cα atoms for 1000 snapshots at 100-ps time intervals. The first two principal components (PC1 and PC2), which are the first two eigenvectors of a covariant matrix, were determined and 2×2 covariance matrices were created using Cartesian coordinates of Cα atoms. The PC plot was constructed by origin software. For further assessment of BCL2's conformational changes after betanin engagement, dynamics cross-correlation matrices analysis (DCCM) was run on the Cα position during the simulations.

Bruncko, M., Oost, T.K., Belli, B.A., Ding, H., Joseph, M.K., Kunzer, A., Martineau, D., McClellan, W.J., Mitten, M., Ng, S.-C., 2007. Studies leading to potent, dual inhibitors of Bcl-2 and Bcl-xL. J Med Chem 50, 641–662.

Lee, T.-S., Cerutti, D.S., Mermelstein, D., Lin, C., LeGrand, S., Giese, T.J., Roitberg, A., Case, D.A., Walker, R.C., York, D.M., 2018. GPU-accelerated molecular dynamics and free energy methods in Amber18: performance enhancements and new features. J Chem Inf Model 58, 2043–2050.

Tuccinardi, T., 2021. What is the current value of MM/PBSA and MM/GBSA methods in drug discovery? Expert Opin Drug Discov 16, 1233–1237.

Ylilauri, M., Pentikäinen, O.T., 2013. MMGBSA as a tool to understand the binding affinities of filamin–peptide interactions. J Chem Inf Model 53, 2626–2633.
